# Pulsed UV Laser Processing of Carbosilane and Silazane Polymers

**DOI:** 10.3390/ma12030372

**Published:** 2019-01-24

**Authors:** Samuel Clark Ligon, Gurdial Blugan, Jakob Kuebler

**Affiliations:** Laboratory for High Performance Ceramics, Empa, Swiss Federal Laboratories for Materials Science and Technology, Ueberlandstrasse 129, 8600 Duebendorf, Switzerland; gurdial.blugan@empa.ch (G.B.); jakob.kuebler@gmail.com (J.L.)

**Keywords:** polymer derived ceramics, silicon carbide, laser processing, pulsed UV lasers, MEMS

## Abstract

Freestanding SiCNO ceramic pieces with sub-mm features were produced by laser crosslinking of carbosilane and silazane polymer precursors followed by pyrolysis in inert atmosphere. Three different pulsed UV laser systems were investigated, and the influence of laser wavelength, operating power and scanning speed were all found to be important. Different photoinitiators were tested for the two lasers operating at 355 nm, while for the 266 nm laser, crosslinking occurred also without photoinitiator. Pre-treatment of glass substrates with fluorinated silanes was found to ease the release of green bodies during solvent development. Polymer crosslinking was observed with all three of the laser systems, as were bubbles, surface charring and in some cases ablation. By focusing the laser beam several millimeters above the surface of the resin, selective polymer crosslinking was observed exclusively.

## 1. Introduction

SiCN and SiCNO ceramic materials are generally known for high thermal conductivity and thermal stability along with chemical inertness and high mechanical strength [1,2,3]. SiC and SiCN ceramics can be formed by sintering of SiC and SiN powders or by pyrolysis of carbosilane and silazane precursors under inert atmosphere [4,5]. Reaction with atmospheric oxygen either during polymer crosslinking or during pyrolysis results in SiCNO. The maximum temperature of pyrolysis and dwell time can be used control micro-structure and concurrently affect the resultant material properties [6]. Liquid precursors of polymer-derived ceramics (PDCs) can be processed by numerous industrially scalable methods including extrusion, molding and film casting [7,8,9]. Lithographic processing of liquid PDC precursors has been investigated by a number of research groups, where it provides some potential advantages compared to lithographic processing of ceramic slurries [10,11,12,13,14,15,16,17]. For instance, light penetration and scattering are problematic in slurries, which limit both processing speed and feature resolution [18].

An array of carbosilane and silazane liquid resins are commercially available, with different rheological properties and cure kinetics to suit the needs of the intended application [8,19]. SMP10 is a commonly used carbosilane precursor with reactive allyl sidegroups. By comparison, Ceraset PSZ 20 is an attractive silazane precursor with vinyl sidegroups [20]. Both precursors can be thermally crosslinked at temperatures below 100 °C with the addition of peroxide initiators or UV crosslinked with an appropriate photoinitiator [19,21,22]. Liew et al. tested three different photoinitiators (Irgacure 651, Irgacure 907, and ITX) to selectively UV crosslink Ceraset and found that a combination of different photoinitiators tended to produce the most perpendicular sidewalls [17]. Kim et al. functionalized different silazane and carbosilane precursors and used different lithographic methods to give green bodies with fine micro-structures [15,23]. More recently, de Hazan used stereolithography to selectively crosslink allyl carbosilane resins with different ratios of acrylate monomers to control nano-porosity [11]. In previous studies in our labs, we used maskless lithography based on a micro-mirror array with filtered Hg-lamp source to produce single and multi-layer SiCN components with sub 100 µm features [24,25]. In this paper, we investigated three different laser systems with rastering scanner heads in an attempt to produce ceramics with similar or possibly better resolution.

Lasers are used to shape ceramics and ceramic composites by both subtractive and additive manufacturing methods [26,27]. In the first case, ablation with pulsed lasers is industrially applied to drill holes in, selectively mill and surface texture various ceramic materials including silicon carbide and silicon nitride [28,29]. Pulsed lasers are also investigated for additive manufacturing of ceramics. The work of Kim et al. on two-photon induced polymerization of both siliazane and carbosilane precursors to yield SiC and SiCN ceramics is worth special mention [30,31,32]. In those studies, they utilized an fs-pulsed Ti:sapphire laser operating at 780 nm to achieve green body feature resolutions below 300 nm. Pulsed Nd:YAG lasers operating at 1064 nm have beam spots 10 times smaller than continuous wave CO_2_ lasers, and thus increased use in the selective laser sintering of ceramic powders was found [33]. Pulsed visible lasers offer even better feature resolution and are also investigated for ceramic processing [34]. While pulsed NIR and visible lasers are actively utilized for additive manufacturing of ceramics, pulsed UV lasers have received less attention [35]. Based on good results on selective laser processing of ceramics with a frequency doubled Nd:YAG laser (532 nm) [36], we chose to investigate frequency tripled (355 nm) and frequency quadrupled (266 nm) pulsed Nd:YAG lasers.

## 2. Materials and Methods

### 2.1. Materials and Instrumentation

Silicon wafers (50.8 mm × 275 µm; Czochralski grown; p-doped) were purchased from Si-Mat (Kaufering, Germany). The carbosilane resin (SMP10) was obtained from Starfire Systems (Glenville, NY, USA). The utilized silazane (Ceraset PSZ 20) was purchased from Clariant Products GmbH (Frankfurt, Germany). Both resins were stored cold in sealed containers prior to use. Silating reagents ((Tridecafluoro-1,1,2,2-tetrahydrooctyl)trichlorosilane (TDFOS) and (3,3,3-Trifluoropropyl)methyldichlorosilane (TFPS)) were purchased from ABCR GmbH (Karlsruhe, Germany). Irgacure 1173 was purchased from BASF (Ludwigshafen, Germany). TPO-L was kindly provided by Rahn AG (Zürich, Switzerland). Unless otherwise specified, chemicals and solvents were used as received without further purification. Photoreactive chemicals and resins were stored in dark polypropylene containers or foil covered glassware and protected from light during transfer, processing and analysis.

UV-vis spectroscopy was performed with a Cary 50 Scan from Varian (Palo Alto, CA, USA) and ATR (attenuated total reflectance) FT-IR using a Bruker Tensor 27 with a diamond tip (Bruker Nano GmbH, Berlin, Germany). Spin coating was performed with a SM-180-BT from Sawatec (Sax, Switzerland) and tape casting with a PA-2325 50 mm adjustable doctor blade from Byk-Gardner GmbH (Geretsried, Germany). Film thickness was generally determined by contact profilometry using an Ambios XP-1 (Ambios Technology, Santa Cruz, CA, USA). In some cases, an optical profilometer was used (an Altiprobe Optic instrument (Cotec Controle Applications, Evian les Bains, France)). Differential scanning calorimetry–thermal gravimetric analysis (DSC-TGA) was performed with a Netzsch STA449 F3 Jupiter instrument (Selb, Germany). A Zeiss Discovery V20 with an external CL 9000 LED light source (Carl Zeiss Microscopy GmbH, Jena, Germany) was used for optical microscopy. Scanning electron microscopy (SEM) was performed with a Tescan VEGA3 (Brno, Czech) with a peripheral Bruker X Flash 6/0 EDX unit (Bruker Nano GmbH, Berlin, Germany). Sputtering was performed on samples immediately prior to SEM by 30-second exposure to a Pt/Pd alloy using a Cressington 108 Sputter Coater (Watford, UK).

### 2.2. Glass Treatment and Coating

Microscopy glass slides (75 mm × 25 mm) were first sonicated 5 min in acetone and then 5 min in isopropanol. After rinsing with deionized water, the slides were sonicated 30 min in 30% KOH and then rinsed well with deionized water. Next, the slides were immersed in a 1M solution of HCl for 30 min, rinsed again with deionized water and dried in an oven for 30 min. The silating solution (2 wt % TDFOS or 2 wt %. TFPS in cyclohexane) was dripped liberally on the front surface of one slide, which was then covered directly with a second slide. After 15 min, the two slides were recoated with silane and re-sandwiched for another 15 min. Both slides were then rinsed with cyclohexane and dried in an oven at 100 °C for 30 min.

For spin coating of films, a solution of 40–50 wt % PDC resin (Ceraset or SMP-10) with 2.5 wt % Irgacure 1173 in m-xylene was prepared and stirred in a dark container for an hour. The resin was applied to untreated Si-wafers (p-doped 1-0-0) and then spun at different speeds. In preliminary experiments to measure film thickness, the films were afterwards placed in a UV oven (BLX-365 from Vilber Lourmat (Merne La Vallee, France) retrofitted with 5 × 8 W 254 nm bulbs) and cured for 10 min (approx. 5 mW cm^−2^). Thicker films were prepared by tape casting. In which case, the desired blade gap was first set from the top surface of the glass slide. In a typical experiment, 200 µL of resin was applied to a slide and manually cast with a speed of approximately 50 mm s^−1^. Photo-crosslinking was performed either non-selectively using a hand-held UV lamp (UVA-Hand from Hoenle AG, Munich, Germany) with an intensity of 120 mW cm^−2^ or selectively using one of three laser systems.

### 2.3. Laser Processing

Three different pulsed UV laser systems were investigated. The first system (266 nm) is based on an Avia 266-3000 laser (Coherent, Inc., Santa Clara, CA, USA) with a pulse length of 25 ns, beam spot size of 5 µm and average output power of 3.0 W at 30 kHz. The laser was equipped with a VA-CB-266-Conex beam attenuator from Newport Optics (Irvine, CA, USA) allowing a reduction in beam intensity of up to 200×. The second system investigated was an Avia 355 nm laser (Coherent, Inc., Santa Clara, CA, USA) with a 20-40 ns pulse length and output of 5.5 W at 40 kHz. The third system was a SuperRapid laser from Lumera GmbH (Kaiserslautern, Germany) with a 10 ps pulse length, pulse rates up to 640 kHz and average power up to 4 W at the wavelength of 355 nm. All three systems were equipped with galvano-scanners from ScanLab (Puchheim, Germany) to selectively raster the beam on the resin surface. Details of experimental conditions used for each laser system are provided within the Results and Discussion section.

### 2.4. Solvent Development

Following laser crosslinking, glass slides with attached green bodies were immersed in a 50:50 mixture of acetone and isopropanol for approximately 30 s to remove non-crosslinked resin. The green bodies were then rinsed with isopropanol for about a min. During this time, the green bodies released on their own from their glass substrates. The pieces were then manually transferred using thin polyethylene film fragments and placed atop a thin nylon mesh to dry.

### 2.5. Pyrolysis

Green bodies formed by laser crosslinking were transported within black polypropylene containers and transferred into flat graphite crucibles. The crucibles were covered and placed horizontally into the central heating zone of a Carbolite tube furnace (STF 16/610) (Sheffield, UK) with heat shields on either end. The atmosphere in the tube was first evacuated with a small vacuum pump and purged twice with argon before heating. Under a steady flow of argon, the samples were heated first to 250 °C and held there for two hours before ramping the temperature to a maximum of 1200 °C. Samples were pyrolyzed at this temperature for two hours and then cooled to ambient. The heating rate was 60 °C hr^-1^ and the cooling rate was 80 °C hr^−1^.

## 3. Results and Discussion

### 3.1. Controlling Carbosilane and Silazane Flim Thickness on Silicon and Glass

Spin coating is an established method for creating thin uniform films. Generally, the higher viscosity of the silazane resin (Ceraset) allows thicker layers than those formed with SMP10. Films were spun on both silicon wafers and on glass slides, since the PDC precursors wet both surfaces very well. Layer thickness is dependent on the concentration of polymer in the solution and on spinning speed. After spin coating, the films were UV cured with a hand-held Hg lamp to give a relatively hard coating. UV flood curing (with a hand-held Hg lamp) is simple and reliable and was thus used in these preliminary experiments to determine the relation between coating conditions and layer thickness. Layer thickness was then determined by contact profilometry (Table 1) and is expressed as an average of five measurements with a standard deviation.

Since 500 rpm is already very slow for spin coating and 50 wt % is a fairly high concentration, the maximum thickness expected for Ceraset films using spin coating is only 10–20 µm. To achieve uniform films with thicknesses greater than 10 µm, tape casting is more appropriate.

Both carbosilane (SMP10) and silazane (Ceraset) precursors are readily amenable to tape casting. Ceraset has a higher viscosity (0.25 Pa·s vs. 0.10 Pa·s @ 25° C with a sheer frequency of 10 Hz), which allows better homogeneity particularly for thick films. After adding 5 wt % photoinitiator (Irgacure 1173), Ceraset was also found to crosslink more rapidly than SMP10 with photoinitiator. This is thought not to be due to a difference in the reactivity of carbosilanes and silazanes, but rather due to the higher reactivity of vinyl groups (in Ceraset) versus the allyl groups (in SMP10). Both resins were sequentially tape cast on glass substrates and UV crosslinked with a hand-held Hg lamp (Table 2).

Sequential coating and curing parameters for both resins were:

1. Casting with a 127 µm blade gap, then 5 min UV curing.
2. Followed by casting with a 254 µm blade gap, then 5 min UV curing.
3. Followed by casting with a 381 µm blade gap, then 5 min UV curing.
4. Followed by casting with a 508 µm blade gap, then 5 min UV curing.

The resultant layer thicknesses measured after each step are summarized in Table 2. The values in the table are the average of five measurements taken at different positions along with a standard deviation. With both formulations, sequential coating and curing allowed thicknesses after four cycles greater than 200 µm. However, the Ceraset films are far more interesting due to better film homogeneity and a stable increase in thickness with each coating cycle.

### 3.2. Glass Treatment to Facilitate Green Body Release

**Figure 1:**
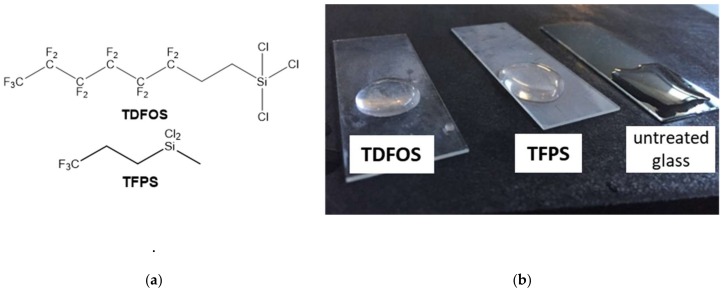
Silane reagents (**a**); water on TDFOS treated glass, TFPS treated glass and untreated glass (**b**).

The PDC green bodies must be removed from the glass or silicon substrate prior to sintering. This is difficult due to non-selective adhesion of silazane precursor to the glass substrate. Thus, the glass was first treated with a fluorinated chlorosilane (TDFOS), which rendered it both hydrophobic and oleophobic. Importantly, Ceraset still wetted the fluorinated glass sufficiently to give homogenous thin films. On the other hand, Ceraset with 20 wt % hexanediol diacrylate (HDDA - added to certain compositions to improve green body mechanical properties) did not wet the TDFOS treated glass. In this case, a shorter chain fluorinated silane (TFPS) was used for substrate pre-treatment. Figure 1 demonstrates wetting behavior of water on the three substrates, where the slide in the middle with a static contact angle between 90–110° is optimal.

### 3.3. Spectroscopic Analysis of SMP10 and Ceraset

**Figure 2:**
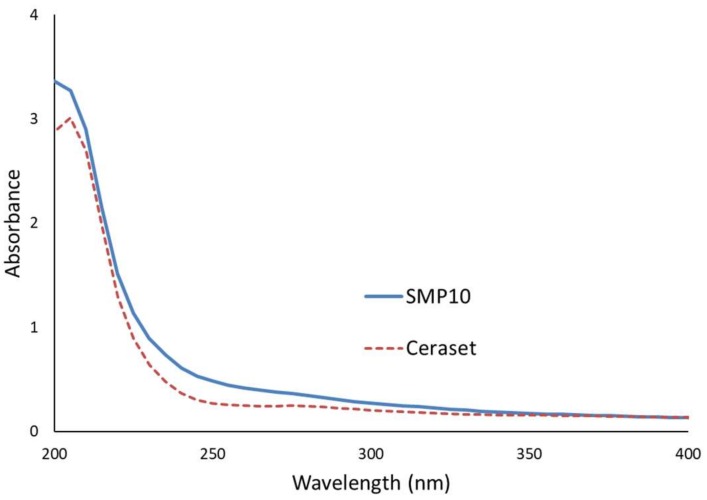
UV-vis absorbance of carbosilane SMP10 and silazane Ceraset (0.05 wt % in c–hexane).

UV-vis spectroscopy was performed on dilute solutions of both SMP10 and Ceraset (0.05 wt % in c–hexane) to determine their suitability for laser processing. As Figure 2 indicates, absorbance is rather low at 355 nm. Below 300 nm, absorbance increases for both resins, in particular for SMP10. For this reason, photoinitiator may not be needed to crosslink with the 266 nm laser system. For the two 355 nm lasers, different photoinitiators that absorb well at this wavelength were added to the resins.

### 3.4. Laser Fabrication

Three different laser systems were investigated for selective crosslinking of carbosilane and silazane resins. For all three systems, objects were produced by creating a hatch that was written by the scan head.

#### 3.4.1. Laser System 1: ns-Pulsed 266 nm

**Figure 3:**
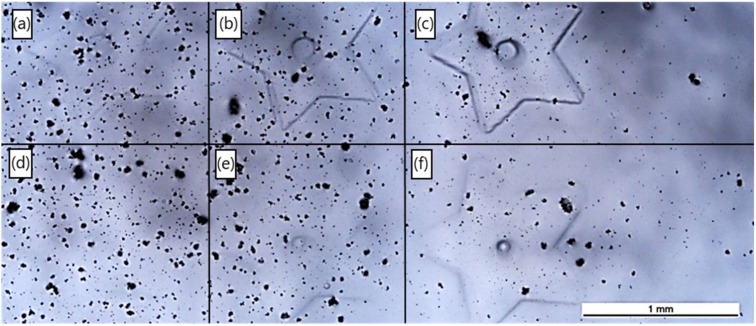
Initial experiments with the 266 nm laser and Ceraset. In the top row (**a–c**), the laser focal point was 6 mm above the surface of the resin. In the bottom row (**d–f**), the laser was 9 mm out of focus. Objects in frames (**a**) and (**d**) were formed with one scan of the star pattern, (**b**) and (**e**) were formed by repeating the pattern four times, and (**c**) and (**f**) were formed with eight repetitions. In all cases, laser power was 0.3 W and hatching distance was 10 µm.

The first laser system studied was a high power Q-switched UV laser operating at 266 nm. This unit can provide up to 3 W average laser output power. Such high power is optimal for material machining and ablation, but is generally too high for photo-crosslinking of polymers. Different methods for reducing laser power were tested. In initial experiments with the 266 nm laser, power was effectively reduced by operating with the laser focal point intentionally set above the working surface. Figure 3 illustrates a few phenomena in using this strategy with Ceraset. First, the black flecks are ablation dust, which was produced when the laser focal point was set on the surface of the resin. With laser power reduced to 0.3 W, ablation still occurred with the laser 3 mm out of focus. By readjusting the laser further out of focus, conditions were achieved where crosslinking, however, no ablation occurred. Star-shaped structures were formed with the laser 6 mm and 9 mm out of focus. The structure was barely visible when the hatch pattern was written only once (Figure 3a,d), and became progressively more visible with four (Figure 3b,e) and eight repetitions (Figure 3c,f). Although selective crosslinking could be demonstrated, the features were not very sharp, as would be expected from working out of focus. 

To further reduce the output power without sacrificing feature resolution, the laser was retrofitted with a power attenuator. The chosen module (VA-CB-266-Conex beam attenuator from Newport Optics) reduces laser power by as much as 200× using a variable beam splitter consisting of a specially designed opto-mechanical adapter with two thin-film Brewster type polarizers. The included software was used to calibrate the transmitted output from the variable beam splitter at the operating wavelength.

**Figure 4:**
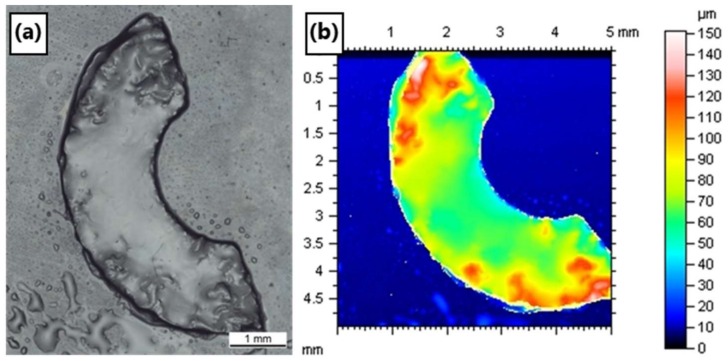
(**a**) Optical microscopy image of soft but freestanding Ceraset green body crosslinked by the 266 nm laser (40 mW power with 10 µm hatching) and (**b**) optical profilometry image.

SMP10 and Ceraset were separately applied to TFPS treated glass slides and spread with a doctor blade to give films of approximately 100 µm. A donut-patterned bmp file was used to define the hatch pattern of the scanner head. With a laser power of 40 mW, crosslinking was noted in both resins. After scanning the resin 10 times, the green bodies were washed with solvent and removed from their substrates. Figure 4 presents the resultant crosslinked Ceraset green body, which ripped during handling. Optical microscopy and profilometry were used to characterize the surface topology and layer thickness. According to the optical profilometry measurements, the green body layer is around 60–80 µm thick.

**Figure 5:**
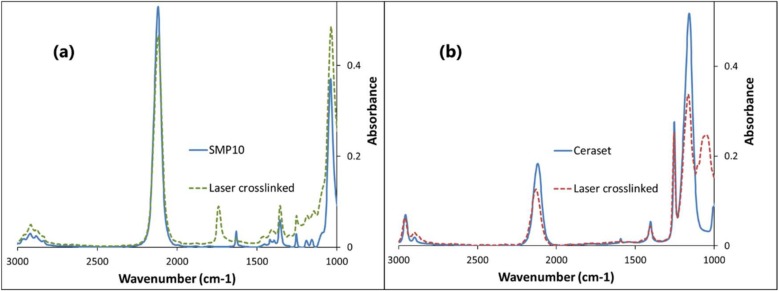
ATR-IR of SMP10 (**a**) and Ceraset (**b**) before (solid blue curves) and after crosslinking (dashed curves).

Green bodies formed by laser crosslinking were examined afterwards by ATR-IR (Figure 5). Both resins have strong peaks at approximately 2120 cm^−1^ corresponding to Si–H stretching. In both cases, this signal decreases with crosslinking although the change is more pronounced with Ceraset. By comparison, the carbon-carbon double bond signal at approximately 1600 cm^-1^ diminishes much more dramatically with SMP10 than with Ceraset. For SMP10, the signal was replaced with a new broad signal at 1700 cm^−1^, which was not seen in the crosslinked Ceraset. This indicates that new C=C and, or C=O bonds have formed. These could arise by reaction of the resin with atmospheric oxygen, where the breadth of the peak is evidence that multiple reaction products have formed. Changes occurring in the fingerprint region are also worth mentioning. The SMP10 resin had two Si–C stretching peaks at 1190 and 1155 cm^−1^. After crosslinking, the peak at 1155 cm^−1^ disappeared, which can be explained by multiple crosslinking processes. The increased signal at 1050 cm^−1^, which was seen for SMP10 but even more so in the crosslinked Ceraset, is most likely Si–O stretching, which indicates that hydrolysis or oxidation are playing significant roles in the laser crosslinking of both resins. These reactions have been noted by other researchers [37] and are quite feasible since laser crosslinking was performed in ambient air with no measures taken to exclude oxygen or water.

**Figure 6:**
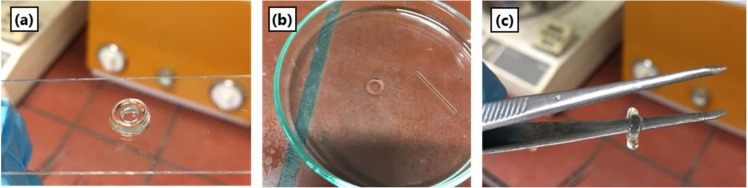
Ceraset green body formed by single layer laser crosslinking (266 nm system with power of 90 mW and hatch width of 2 µm). (**a**) Green body after solvent wash; (**b**) demonstrated removal from substrate; (**c**) demonstrated mechanical stability.

Processing conditions with the 266 nm laser, including power and hatching distance, were adjusted to determine those best to give thick and stable green bodies. As Table 3 points out, it was possible to form Ceraset green bodies with thicknesses of 1 mm when hatching distance was lowered to 2 µm and the power was reduced below 100 mW. At higher power, bubbles formed. The disadvantage of low hatching width is longer scan times (approximately 3 min per scan for test three or 15 min for five repetitions). By comparison, thinner green bodies could theoretically be formed in less time per layer, however, this was effectively much longer due to time for recoating. Moreover, thick single layer green bodies were found to be mechanically more robust than multi-layer green bodies formed with the laser (formed by recoating 100 µm thick layers). Although photoinitators were also tested for both resins with the 266 nm laser (Irgacure 1173 and methyl benzoyl formate), the best results were found with Ceraset alone (Figure 6).

#### 3.4.2. Laser System 2: ns-Pulsed 355 nm

**Figure 7:**
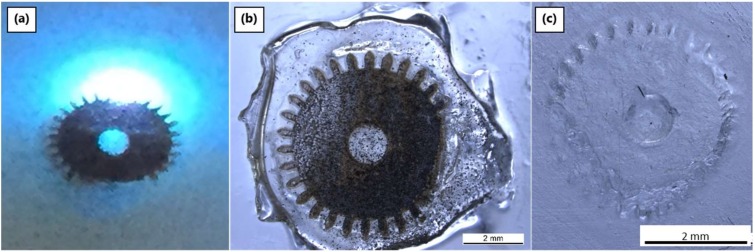
Experiments with ns-pulsed 355 nm laser: (**a**) during laser scanning with 150 mW; (**b**) resultant crosslinked polymer with black ablated pattern; (**c**) photo-crosslinked green body (using 50 mW power) without ablation.

The second laser system investigated has a wavelength of 355 nm and adjustable pulse length in the nanosecond realm. Ceraset resin with 5 wt % TPO-L was cast on TFPS treated glass substrates and placed in the working plane of the laser. Laser power was varied from 150 mW down to 5 mW, while pulse length, scan speed and number of scans were adjusted as well. In initial tests with the pulse length set to 30 ns, ablation occurred (Figure 7a). As Figure 7b shows, heat diffusion from laser ablation caused gelation in the peripheral polymer at distances of more than 2 mm. After adjusting the operating frequency and consequently lengthening the pulses to 40 ns, polymer crosslinking was observed without ablation. The best results (entry 6 in Table 4) were found with a pulse length of 40 ns, a power of 80 mW and scan speed of 50 mm s^−1^. Non-specific crosslinking from heat remained a problem. At lower powers (< 20 mW) or with too rapid scanning speeds (500 mm s^−1^), no crosslinking was observed even with more than 10 scans.

#### 3.4.3. Laser System 3: ps-Pulsed 355 nm 

The third investigated laser system also operated at 355 nm, but with a significantly shorter pulsewidth (10 ps). As with laser system 2, the photoinitiator that absorbs well at this wavelength (TPO-L) was added to the resin (Ceraset) prior to tape casting and processing. Laser power was reduced to 32 mW, since below this, the beam tended not to be stable. As had been observed with the other laser systems, ablation was the principal reaction when the laser was used in focus. As the laser focal point was moved away from the working surface, a mix of ablation and crosslinking was observed. This became exclusively crosslinking when the laser was focused a few mm above the plane of the silazane resin. 

**Figure 8:**
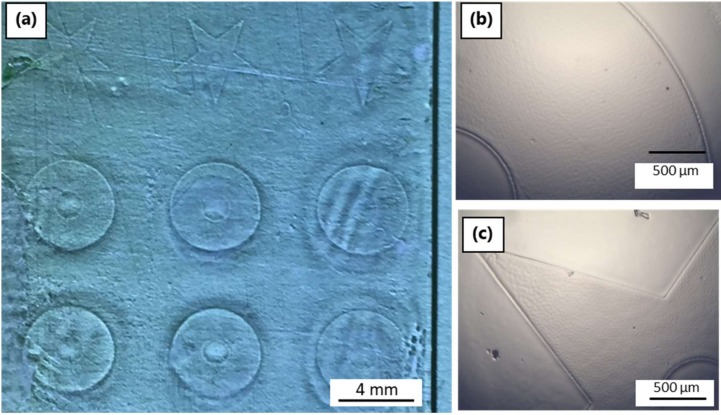
Laser patterning of SMP10/Ceraset with a 355 nm ps-pulsed laser: (**a**) after laser crosslinking and prior to solvent development, the first column of structures was formed with one scan, the second column with two scans and the third column with four scans. Following development: (**b**) 100 µm thick disc structure and (**c**) 40 µm thick star structure.

One of the principle questions to be answered with all of the laser systems was how deep the PDC resin could be crosslinked. To answer this question, a mixture of Ceraset and SMP10 with photoinitiator (3 wt % TPO-L) was coated at different thicknesses on glass slides and selectively patterned with the laser. The experiment used two test shapes: a 5 mm star with a 1 mm central circle and 5 mm discs with 1 mm internal circles. With both shapes, laser focus was adjusted as was the number of scans. For the production of discs, two different rastering styles were used: for the discs in row 2 (Figure 8a), the beam followed a spiral path and with the other rows (Figure 8a), the laser beam was swept linearly in the x-direction followed by y-steps (x-y hatching). After patterning a layer of resin, the sample was washed with a mixture of acetone and isopropanol to remove the unexposed regions. Generally, the disc patterns survived solvent washing much better than the stars. For instance, discs with thicknesses up to 100 µm were possible (Figure 8b), while stars with thicknesses from 10 to 40 µm could be produced (Figure 8c). Scanning the pattern multiple times within the resin provided superior results. More surprisingly, the discs created by x-y rastering were more homogenous and stable than those formed by spiral scanning. The reason for this result is unclear, since the spiral rastering took longer than the x-y raster and thus a higher dose of photons was used for the same volume of resin.

**Figure 9:**
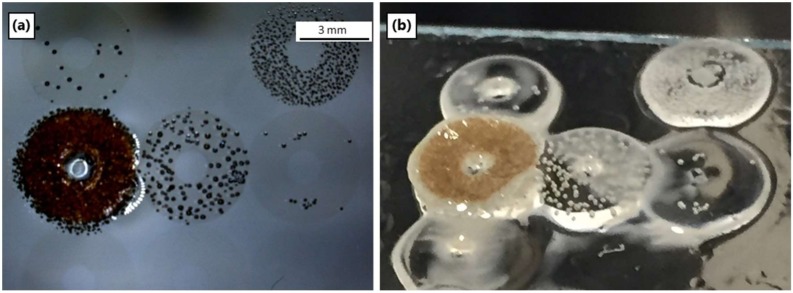
Ceraset green bodies via laser crosslinking at 355 nm: (**a**) before and (**b**) after solvent washing. For the top row, laser power was 130 mW with focus off by 3 mm for the left disc and 1 mm for the disc on the right. The second and third rows of discs were formed with the laser focus 5 mm and 10 mm above the sample. Laser power for both rows was decreased from left to right 560 mW, 400 mW, 280 mW. In the third row, only the first disc survived solvent washing.

After obtaining good results with Ceraset alone using the 266 nm laser, the resin was tested again with the 355 nm ps-pulsed laser. In this case, Ceraset with 0.5 wt % TPO-L was cast with a thickness of 700 µm on an untreated glass substrate. Differences in both optics and pulse systems did not allow direct comparison of laser power for the two systems. Laser power density was adjusted by sequentially moving the focal point of the beam away from the working surface to determine optimal conditions for crosslinking. Generally, crosslinking was preferential at lower power and with increased scan numbers. As the focal point of the beam was moved closer, bubbles formed, and at even higher power, discoloration on the upper surface of the resin occurred (Figure 9). After solvent development of the green bodies, all of the discs, including the discolored ones, were found to be rather soft, which complicated further processing. 

### 3.5. Thermal Analysis

**Figure 10:**
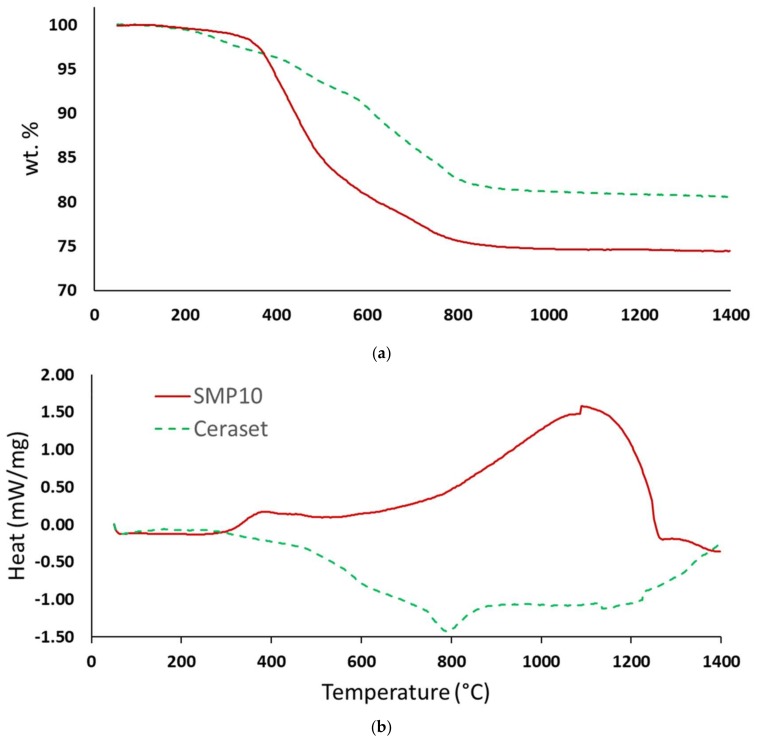
TGA (**a**) and DSC (**b**) of UV laser crosslinked SMP10 (solid red curves) and Ceraset (dashed green curves).

Following laser crosslinking, green bodies were subjected to thermal gravimetric analysis with coupled calorimetry (DSC-TGA) to find suitable conditions for pyrolysis. As Figure 10 shows, weight loss is essentially complete by 800 °C for both materials. SMP10 has a weight loss of about 25% while Ceraset loses only 20%. The DSC curves are, by comparison, quite different. Ceraset exhibits a weak exotherm, which diminishes beyond 300 °C. SMP10, on the other hand, appears unreactive at low temperature and starts to behave exothermically above 350 °C. This exotherm becomes progressively stronger from 700 to 1100 °C. This first exotherm is from crosslinking of residual allyl groups, while at higher temperatures, burn out of hydrogen and small molecule hydrocarbons is also occurring. Ceraset has a lower portion of both carbon and hydrogen and consequently displays smaller exotherms at these temperatures. The slight exotherm beyond 1300 °C could be due to reaction with N_2_ gas to give silicon nitride. Although N_2_ was used for DSC-TGA analysis, pyrolysis of green bodies was performed with high purity argon.

### 3.6. Pyrolysis of Ceraset Green Bodies

**Figure 11:**
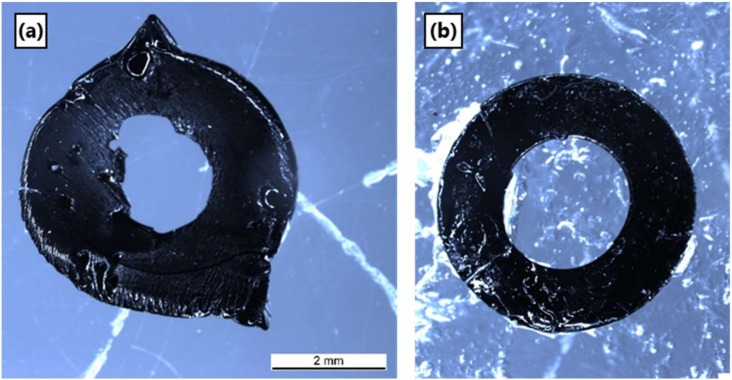
SiCN ceramics sintered with slow heating/ cooling ramps alone (**a**) and with an additional pre-sinter thermal crosslinking step (**b**).

Attempts to pyrolyze thin (100–300 µm) multi-layer green bodies from SMP10 and from Ceraset were unsuccessful due to their poor mechanical properties. Thicker (> 500 µm) single layer green bodies, by comparison, were easier to handle and could be further processed. Ceraset green body discs formed via laser crosslinking with the 266 nm laser system were transferred to graphite crucibles for pyrolysis. Thin donut shaped pieces were heated to 1200 °C in argon and were found to shrink fairly isotropically. Thickness and diameter (internal and external) were measured before and after pyrolysis and a volumetric change of 40% was determined. In a subsequent pyrolysis experiment, it was noted that the resultant ceramic discs were warped. Whether warpage had occurred during heating or cooling was yet to be determined. Multiple strategies for reducing warpage were found in the literature and tested. One method for countering shrinkage, covering the green body with a graphite mesh to provide a counter pressure, resulted in all three cases in catastrophic failure [38]. A successful method, on the other hand, was to include a 2-hour thermal crosslinking step at 250 °C prior to ramping to the sintering temperature of 1200 °C. Figure 11 shows ceramic discs formed via slow heating and cooling ramps alone (a) and discs processed with an additional thermal crosslinking step prior to sintering (b). 

### 3.7. SEM and Microstructure

**Figure 12:**
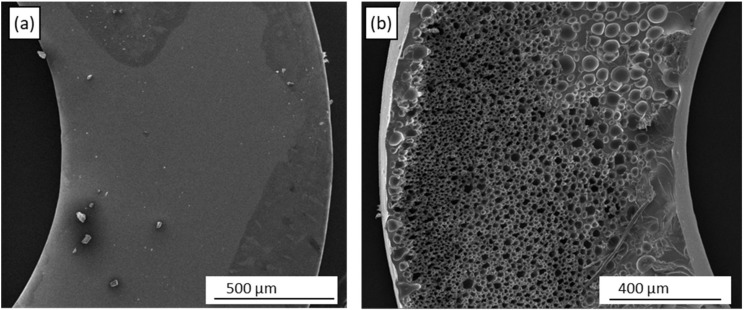
Scanning electron microscopy (SEM) images of front (**a**) and back (**b**) surfaces of SiCNO ceramics formed by pyrolysis of laser crosslinked Ceraset. The microporosity in (**b**) stems from oleic acid, which was used to prevent adhesion during laser processing.

Scanning electron microscopy (SEM) was performed on the top and bottom surfaces of the flat discs (5 mm × 0.5 mm) formed by pyrolysis of laser-crosslinked Ceraset. As seen in Figure 12a, the top surface of the disc is dense and essentially featureless. The bottom surface, on the other hand, displays extensive microporosity (Figure 12b). Similar morphology has been seen by other researchers when they included an organic porogen within the precursor resin [39,40,41,42]. In our case, it was noted that oleic acid had been used during laser crosslinking of the piece to prevent adhesion to the substrate. Conceivably, the oleic acid phase separated on the surface of the resin during crosslinking and was afterwards either washed out during solvent development or burned out during pyrolysis. This type of morphology was not seen in subsequent ceramic pieces produced without oleic acid. Since porosity was not the intent of our project, these results were not further investigated. EDX of the samples indicates an elemental composition of SiCNO with approximately 10-15 atomic % oxygen. As seen by FTIR (Figure 5b), some portion of this oxygen was introduced during polymer crosslinking. Although care was taken to exclude oxygen during pyrolysis (see Experimental Section), one cannot rule out that some portion was also introduced in this step.

## 4. Conclusions

Freestanding single layer SiCNO ceramic pieces with sub-mm features have been produced by laser crosslinking of Ceraset resin followed by pyrolysis. Three different pulsed UV laser systems were tested and it was found that output power and hatching parameters had strong influence on polymer crosslinking and on feature resolution. Excessive heat was an issue with all three laser systems, and focusing the laser several mm above the resin surface did help to alleviate this problem, although structure resolution was compromised. After fitting the system with a beam attenuator, the ns-pulsed 266 nm laser gave the most promising results. Fitting the two other laser systems with beam attenuators was not feasible, which complicated a direct comparison of the systems and made it difficult to better discern the role of wavelength. Nevertheless, green bodies with thicknesses from 500 µm to more than 1 mm could be formed with the 266 nm laser in a single laser-processing step. Crosslinking was improved by scanning multiple (> 10) times. The resultant green bodies were mechanically robust and could be washed with organic solvent. Shrinkage incurred during pyrolysis to SiCNO ceramics was about 20% linearly and was fairly isotropic. These findings should help to improve understanding of laser processing of PDC materials for the fabrication of MEMS components.

## Figures and Tables

**Table 1:** Thickness of spin coated Ceraset films.

Formulation	Substrate	Speed (rpm)	Thickness (µm)
50% in xylene	silicon	1000	3.80 ± 0.03
50% in xylene	silicon	500	5.80 ± 0.02
50% in xylene	glass	500	5.90 ± 0.16

**Table 2:** Thickness of tape cast films after multiple coat/cure cycles. UV curing was with 120 mW cm^−2^ lamp.

Blade Height	Ceraset & 5 wt % PI	SMP10 & 5 wt % PI
(µm)	Thickness (µm)	Thickness (µm)
127	70.0 ± 0.5	65 ± 24
254	140 ± 4	120 ± 42
381	210 ± 2	198 ± 40
508	280 ± 5	247 ± 44

**Table 3:** Processing conditions tested with system 1: 266 nm laser. Entry 5 is highlighted to indicate best results.

Test	Power (mW)	Hatching (µm)	Repetitions	Thickness (µm)	Comments
1	300	10	1	50	partial ablation
2	40	10	10	70	stable green body
3	150	5	5	200	poor crosslinking
4	150	2	5	1000	crosslinking; bubbles
5	90	2	5	1000	stable green body

**Table 4:** Processing conditions tested with laser system 2: 355 nm ns-pulsed laser. Entry 6 is highlighted to indicate the best results.

Test	Power (mW)	Pulse Length (ns)	Scan Speed (mm s^−1^)	Repetitions	Comments
1	150	30	20	1	ablation
2	150	40	20	1	crosslinking; poor resolution
3	150	40	100	5	crosslinking; decent resolution
4	150	40	500	10	no crosslinking
5	80	40	50	10	crosslinking; poor resolution
6	50	40	50	10	crosslinking; better resolution
7	35	40	50	10	insufficient crosslinking
8	15	40	50	10	no crosslinking
